# Psychopharmacological treatment of disruptive behavior in youths: systematic review and network meta-analysis

**DOI:** 10.1038/s41598-023-33979-2

**Published:** 2023-04-28

**Authors:** Ji-Woo Seok, Brigette Soltis-Vaughan, Brandon J. Lew, Aatiya Ahmad, R. J. R. Blair, Soonjo Hwang

**Affiliations:** 1 Department of Psychiatry, University of Nebraska Medical Center, 985578 Nebraska Medical Center, Omaha, NE 68198-5578 USA; 2 Digital Health Research Division, Korea Institute of Oriental Medicine, Daejeon, South Korea; 3 Child and Adolescent Mental Health Centre, Mental Health Services, Capital Region of Denmark, Copenhagen, Denmark

**Keywords:** Medical research, Paediatric research

## Abstract

To conduct a systematic review of the comparative efficacy of various psychotropic medications for the treatment of disruptive behavior (DBs) in youths. To this aim, we systematically reviewed randomized clinical trials (RCTs) of various psychotropic medications targeting symptoms of DBs and applied network meta-analysis to investigate their relative efficacy. Fifty-five RCTs meeting the inclusion criteria were selected. To predict and interpret relative treatment efficacy, we compared the efficacy of various psychotropic medications prescribed for DB symptoms based on their mechanism of action. Network meta-analysis revealed that for reducing DBs, second-generation antipsychotics, stimulants, and non-stimulant ADHD medications were more efficacious than placebo, and second-generation antipsychotics were the most efficacious. The dopaminergic modulation of top-down inhibitory process by these medications is discussed in this review. This study offers information on the relative efficacy of various psychotropic medications for the treatment of DB, and insight into a potential neurobiological underpinning for those symptoms. It also illustrates the potential utility of these neurobiological mechanisms as a target for future treatment studies.

## Introduction

Disruptive behavior problems (DBs) in children and adolescents are one of the most common reasons for referral to mental health care facilities^1^. They are a primary component of serious child and adolescent psychopathologies^2^, and are characterized by behavioral manifestations that typically share difficulties in modulation of aggression, self-control, and impulsivity, and also result in threats to the safety of others and disrupt social norms^3,4^. Oppositional, aggressive, and hyperactive behaviors present in early childhood often predict negative mental health outcomes later in life, ranging from school failure to substance abuse and criminality^5,6^.

For complex disorders with limited existing treatment options and interventions with relatively lower efficacy/effectiveness, clinicians and researchers have attempted to identify subgroups of individuals based on clusters of specific symptoms or other clinical and/or socio-environmental factors^7^. The utility of identifying subgroups is unfortunately limited by the methods used, such as using a categorical diagnostic approach^8^. Because individuals with the same categorical diagnosis display a wide range of symptoms, groups characterized in this manner are very heterogeneous^9^. Furthermore, youth with multiple comorbid disorders are more common in child and adolescent psychiatry^10^.

Recent clinical research has shifted interest to shared transdiagnostic psychopathologies, in accordance with the dimensional model for common psychiatric disorders^11^. Prior studies have demonstrated that DBs are associated with almost all forms of psychopathology in children and adolescents and are broadly present alongside both internalizing and externalizing symptoms^12–14^. Therefore, DBs may be excellent candidates as trans-diagnostic markers for studying the effectiveness of interventions, as well as guide ongoing and future child and adolescent psychiatric research and clinical practice^15^.

Use of a transdiagnostic method is further supported by the fact that there may be a common underlying pathophysiological mechanism of DBs in youths across various psychiatric diagnoses. Impairments in emotion regulation and inhibitory control^16–19^ may be key predictors of DBs^20,21^. According to the dysregulation hypothesis, DBs can be induced by the failure of top-down modulation of neural areas implicated in emotional responding^16^. This model possibly implicates the mesolimbic dopamine system, including projections from the ventral tegmental area to the nucleus accumbens and ventral striatum, and eventually to the prefrontal cortex^22–24^. Additionally, previous research has suggested that DBs in various psychiatric diagnoses result from dysfunction in the serotonergic and dopaminergic systems in the prefrontal cortex^25–27^.

Regarding psychopharmacological interventions, a number of agents have been studied for treatment of DBs in children and adolescents. Prior clinical trials investigated the efficacy of stimulants^28–31^, atomoxetine^28,32–34^, atypical antipsychotics^30,35–40^, mood stabilizers^39,41^, and anticonvulsants^42,43^ for treatment of DBs in youths with various psychiatric diagnoses (i.e., Attention Deficit Hyperactivity Disorder (ADHD), Conduct Disorder (CD), Oppositional Defiant Disorder (ODD), Disruptive Mood Dysregulation Disorder (DMDD), Major Depressive Disorders (MDD), Anxiety Disorders, Autism Spectrum Disorder (ASD), and tic disorder). Most of the previous studies, however, focused on the primary symptoms of each categorical diagnoses, for example, inattention in ADHD, or global improvement, and reported on DBs as co-occurring symptoms. Additionally, several systematic reviews reported an overview of the efficacy and safety of common medications for DBs^44–46^.

The previous studies leave a general gap in knowledge on the relative efficacy of each agent in the treatment of DBs, especially in regard to the different mechanisms of action of medications and their potential impact on the neurobiology of DBs. These studies did not use an objective appraisal of the evidence with a thorough methodological approach^47^, and many of them compared only two interventions (i.e., placebo vs pharmacological intervention), in which a conventional pair-wise meta-analysis may be conducted^48^.

We have instead applied network meta-analysis (NMA)^49^. Unlike traditional meta-analyses, which only allow for direct comparisons of interventions using the pooled data from clinical trials with similar treatment arms, NMA enables the estimation of relative effects of interventions without direct comparison^50^. Specifically, NMA is a method for comparison among multiple treatments simultaneously in a single analysis by integrating direct and indirect data from randomized controlled trials in a network. NMA has become a focus of clinical research since it may help examine the comparative efficacy of several treatments often utilized in clinical practice simultaneously^49^.

To this end, we systematically reviewed randomized trials (55 in total) of various psychopharmacological agents targeting the symptoms that represent DBs (i.e., aggression, hostility, impulsivity, conduct problems, and oppositional defiant problems) and applied NMA to determine their relative efficacy. We included the previous studies of psychotropic agents across medication classes (stimulants, anticonvulsants, second-generation antipsychotics, selective serotonin reuptake inhibitors (SSRIs), aripiprazole, tricyclic antidepressants, non-stimulant ADHD medication, lithium, and dasotraline), which contain valid measures for DBs even though the treatment of DBs may not have been a primary outcome.

## Methods

### Study protocol registration

This study was written in compliance with the recommendation of the Preferred Reporting Items for Systematic Reviews and Meta-Analyses (PRISMA) statement^51^. The study protocol was registered with the International Prospective Register of Systematic Reviews (PROSPERO) in February 2022 (CRD42021256959). Supplementary Table 1 reported our results based on the updated PRISMA checklist for NMA.

### Search strategy and study selection

We applied a comprehensive search strategy in literature databases. Six electronic databases (PubMed, Cochrane Central Register of Controlled Trials (CENTRAL), EMBASE, Web of Science, PsycINFO, and ProQuest Dissertations) were used for literature search and review for articles published up to February 2022 with the following search terms: (disruptive behavior OR conduct problem OR oppositional problem OR defiant problem OR aggression OR hostility OR impulsivity) AND (children OR adolescent OR youth) AND (randomized OR random OR randomly OR randomization OR randomization OR RCT OR RCTS) AND (pharmacological treatment OR pharmacological therapy OR pharmacological intervention OR drug OR medication OR medication treatment OR norepinephrine-dopamine reuptake inhibitors (methylphenidate stimulants, amphetamine stimulants, OR bupropion) OR dopamine receptor antagonists (aripiprazole, haloperidol, ziprasidone, OR ecopipam) OR GABA receptor agonists (divalproex sodium, valproate sodium, divalproex, diazepam, zolpidem, OR carbamazepine) OR selective norepinephrine reuptake inhibitors (atomoxetine) OR serotonin-norepinephrine inhibitors (duloxetine, desvenlafaxine, nortriptyline, clomipramine, imipramine, OR venlafaxine) OR serotonin-dopamine antagonists (risperidone, lurasidone, clozapine, OR quetiapine) OR selective serotonin reuptake inhibitors (sertraline, paroxetine, escitalopram, fluvoxamine, trazodone, OR fluoxetine) OR Serotonin-norepinephrine-dopamine reuptake inhibitor (dasotraline) OR lithium. After the initial search and review, a manual search was performed by reviewing the reference lists of all identified publications and reviewing similar articles suggested by the meta-analysis and systematic review articles relevant to this topic^52^. To supplement incomplete reports in the original papers, relevant authors were contacted.

### Selection criteria and full-text screening

The review process identified randomized clinical trials (RCTs) assessing the efficacy of psychotropic medications on disruptive behavior. All RCTs of pharmacological treatment reporting measures of symptoms that may represent underlying disruptive behavior were considered for inclusion. Based on our literature review^3,53^, the following symptoms were selected because they may represent underlying DB: (1) aggression/hostility, (2) oppositional/defiant problem, (3) conduct problem and (4) impulsivity. For the measurements that were used for each symptom, see Table 2.

The following exclusion criteria were applied: (1) abstract-only articles, case reports and case series (2) overlapping data set (3) animal studies (4) studies which did not report the mean or standard deviation in the post-treatment measurements (5) studies investigating comparison of efficacy between psychotropic medications with similar enough mechanism of drug action (i.e., risperidone vs. quetiapine vs. clozapine or dextroamphetamine vs. methylphenidate vs. bupropion) (6) studies of combination of more than one psychotropic medication, as it is difficult to distinguish the efficacy of psychotropic medication from that of combined effect (7) studies using unreliable measurement and studies with unclear data which included non-peer-reviewed publication. There were no limitations on the language, year of publication, country, gender, or ethnicity of the patients. Through the predefined eligibility criteria, two independent reviewers first screened and chose the title and abstract, then conducted full-text screening of included articles. Any disagreement between the reviewers was decided by an independent experienced literature reviewer.

### Data extraction

A standardized template file was created based on a pilot extraction with the two most relevant references. Two researchers separately extracted the data using this template file. Extracted data included: title, authors, publication year, and participants’ characteristics including age, gender, sample size and diagnosis. The data for the dosages of each psychotropic medication, methods of administration, trial duration, measurements for symptoms representing underlying DB, and pre- and post-treatment scores for symptoms were also extracted. The data extracted was reviewed by the two independent researchers and disagreements were resolved through discussion to reach a consensus.

### Risk of bias assessment

Two reviewers independently evaluated the validity of each RCT through “The Cochrane Risk of Bias Tool for randomized controlled trials”^54^. The tool evaluates RCT using a set of domains of bias including random sequence generation, allocation concealment, selective reporting, blinding of participants and personnel, blinding of outcome assessment, incomplete outcome data, and other sources of bias. Each study domain was assigned a rating of low, high, or unclear risk. Any discrepancies were resolved by a discussion among reviewers or guidance from an independent experienced literature reviewer.

### Summary measure

#### Statistical analysis

As pre-specified in the study protocol, we analyzed psychotropic medications according to their primary mechanisms of action. This led to categorization of the listed psychotropic medications as follows: (1) Norepinephrine and dopamine reuptake inhibitors (stimulants), (2) GABA receptor agonists (anticonvulsants), (3) serotonin-dopamine antagonists (second-generation antipsychotics), (4) selective serotonin reuptake inhibitors (SSRIs), (5) dopamine receptor modulator (aripiprazole), (6) Serotonin-Norepinephrine Reuptake inhibitors (Tricyclic antidepressants, TCA), (7) selective norepinephrine reuptake inhibitors (non-stimulant ADHD medication such as guanfacine and clonidine), (8) serotonin-norepinephrine-dopamine reuptake inhibitor (dasotraline) and (9) lithium. The list of medications in each category is provided in Table 1. After the categorization, NMA was performed to compare the efficacy of different psychotropic medications with a frequent random-effects model, which preserves randomized treatment comparisons within trials using the net-meta R package version 8.0 (available at: http://CRAN.R-project.org/package=netmeta)^55^. International Society for Pharmacoeconomics and Outcome Research (ISPOR) recommend the NMA for comparing efficacy between different treatment modalities^56^. NMA enables simultaneous comparisons between all treatment arms across the studies in a single analysis by combining both direct and indirect comparisons^57^.

**Table 1:** The list of medications in each category in our study.

Agent group	Drug lists
Norepinephrine and dopamine reuptake inhibitors (stimulants)	Methylphenidate
GABA receptor agonists (anticonvulsants)	Carbamazepine, Valproate sodium, Divalproex
Serotonin-dopamine antagonists (second-generation antipsychotics)	Risperidone, Quetiapine, Olanzapine
Selective serotonin reuptake inhibitors (SSRIs)	Fluoxetine, Sertraline
Dopamine receptor modulator (aripiprazole)	Aripiprazole
Serotonin-Norepinephrine Reuptake inhibitors (Tricyclic antidepressants, TCA)	Imipramine, Desipramine
Selective norepinephrine reuptake inhibitors (non-stimulant ADHD medication)	Atomoxetine
Lithium	lithium
Serotonin-norepinephrine-dopamine reuptake inhibitor (dasotraline)	Dasotraline

Heterogeneity is estimated by the I^2^ and Q statistics, which measure the percentage of total variation in point estimates among studies that is attributable to heterogeneity^55,58^. The I^2^ statistic describes the percentage of variation that is not attributable to chance. A value of I^2^ from 0 to 50% was considered unlikely to be important, 50–75% as moderate heterogeneity, and 75–90% as considerable heterogeneity^59^. Also, the Q statistics, which are a statistic for inconsistency, are calculated using the weighted sum of squared differences across studies, which represents the variation of treatment effect between direct and indirect comparisons at the meta-analytic level^60^. Significant heterogeneity was examined to be present when the *p* for heterogeneity was < 0.05 in the result of Q statistics.

To assess inconsistency in the network, a net-heat plot was used^61^. The net-heat plot is helpful to examine direct comparisons that might be likely sources of important inconsistency in the network. In that graph, the larger the gray box, the more important that treatment comparison is relative to another treatment comparison and the colored backgrounds indicate the degree of inconsistency in the network^61^. Additionally, the node-splitting method was used for assessment of inconsistency in the forest plots of NMA^62^. Publication bias was assessed by adjusted funnel plots. If data were sufficient, an Egger’s test of the intercept with centralized effect size and SE was further conducted^63^.

Since we assumed that the included studies differed with respect to clinical and other factors, we applied random-effects models instead of fixed-effect models. To rank the treatments based on their efficacy for each outcome, P-scores, a statistical parameter in frequentist NMA and ranging from 0 to 1, were calculated using the surface under the cumulative ranking curve (SUCRA)^64^. A higher P-score expresses the probability that the treatment would be better than the competing treatments^65^.

To interpret frequentist NMA, we checked the confidence interval to find any significant difference between the treatment arm and placebo. Then, we ordered the treatment using P-score.

## Results

### Search results

**Figure 1:**
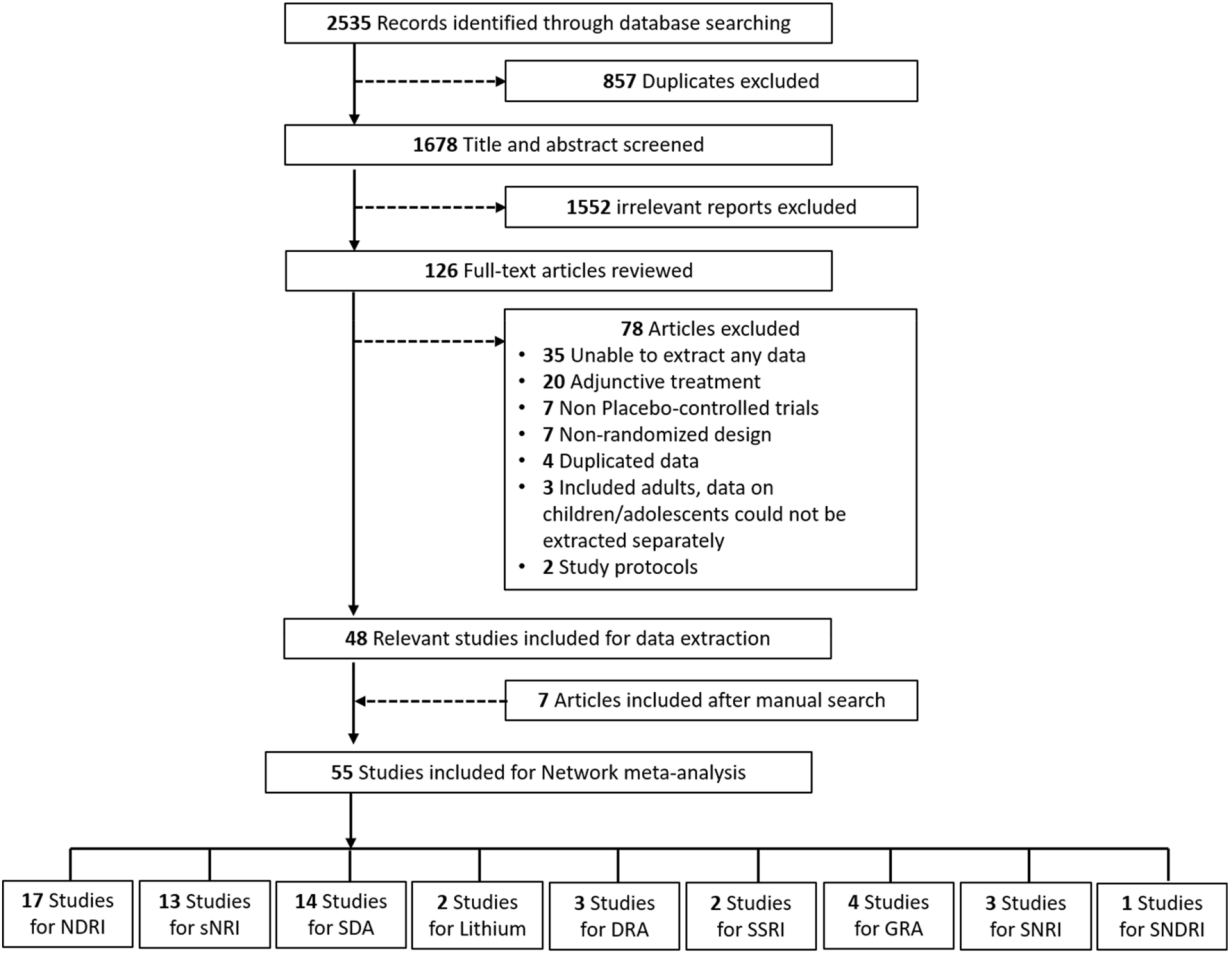
Flow diagram of study selection.

**Figure 2:**
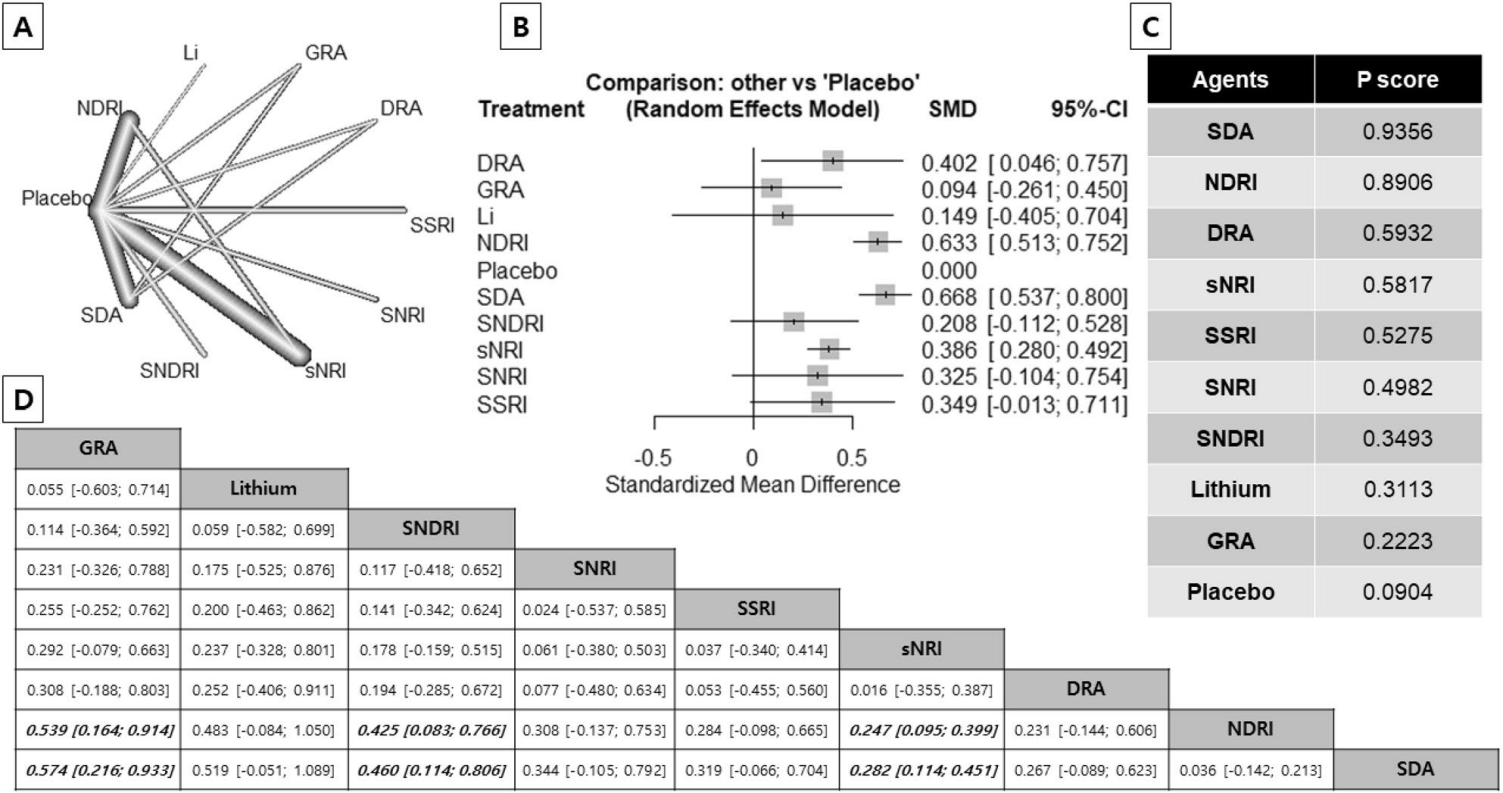
The result of NMA for disruptive symptoms. (**A**) The network graph representing treatment arms included in the network for disruptive symptoms, the thickness of the lines shows the number of studies. (**B**) Random effect model forest plot for comparison of each treatment arm vs. placebo. (**C**) Ranking of medications for disruptive symptoms using SUCRA values. (**D**) Comparison of the included agent groups: standardized mean differences (95% CI). Each cell represents the effect of the column-defining agent group compared to the row-defining agent group. *DRA* Dopamine receptor antagonist/aripiprazole, *NDRI* Norepinephrine–dopamine reuptake inhibitor/stimulant, *SDA* Serotonin dopamine antagonist/second-generation antipsychotics, *sNRI* Selective norepinephrine reuptake inhibitors/non-stimulant ADHD medication, *SNDRI* Serotonin-norepinephrine-dopamine reuptake inhibitor, *SNRI* Serotonin-norepinephrine reuptake inhibitors/tricyclic antidepressants, *SSRI* selective serotonin reuptake inhibitor.

Our initial search yielded 1678 papers relative to our research terms. Title/abstract screening excluded 1552 irrelevant papers while a further 78 papers were excluded following full-text screening due to unavailability of data extraction, presence of adjunctive treatment, etc. Seven additional papers were included from the manual search. In total, 55 RCTs (comprising 5684 patients) were included for the NMA (Fig. 1). In addition, there were four studies comparing individual agent arms against each other (stimulant vs. non-stimulant ADHD medication, second-generation antipsychotic vs. dopamine receptor modulator, and second-generation antipsychotic vs. anticonvulsant) (Table 2, Fig. 2A). For the list of the articles included, see Table 2.

**Table 2:** Studies measuring the symptoms of disruptive behavior problem.

Study	Drug(s)	Dosage	Diagnosis	n in drug group	n in PBO group	Age	Duration (day)	Assessment	DBP mean score (SD) at pre-treatment		DBP mean score (SD) at post-treatment	
									Drug	PBO	Drug	PBO
NDRI												
Bukstein, 1998^101^	MPH vs. PBO	0.6 mg/kg	ADHD	18	18	6–12	21	Overt aggression Scale	–	–	0.27 (0.51)	1.01 (0.99)
Coghill, 2007^29^	MPH vs. PBO	0.6 or 1.2 mg/kg	ADHD	25	25	7–15	84	Restless/impulsive subscale in Conners’ Parent Rating Scale	–	–	68.4 (13.8)	77.1 (1.9)
Evans, 2001^102^	MPH vs. PBO	10, 20 or 30 mg	ADHD	135	45	13.8 (1.2)	42	Oppositional/Defiant subscale in IOWA Conners rating scales	6.0 (5.2)		0.93 (1.78)	2.5 (3.4)
Findling, 2007^103^	MPH vs. PBO	5, 10 or 15 mg/day	DBD	16	16	5–17	28	Conduct Problem in Conners’ Parent Rating Scale	81.4 (13.7)		60.2 (15.4)	73.9 (19.4)
Gorman, 2006^104^	MPH vs. PBO	0.94 ± 0.02 mg/kg	ADHD	40	40	6–12	42	Aggression in inattention/overactivity with aggression	0.55 (0.54)		0.47 (0.36)	0.63 (0.43)
Handen, 2000^105^	MPH vs. PBO	0.3 or 0.6 mg/kg	ADHD, ASD	12	12	5–11	7	Aggression subscale in IOWA Conners Teacher Rating Scale	–	–	2.5 (1.38)	5.75 (4.22)
Huang, 2021^106^	MPH vs. PBO	22, 33 or 44 mg	ADHD	99	99	6–18	14	Hyperactive/Impulsive subscales in Swanson, Nolan, and Pelham Rating Scale-Revised	6.3 (2.7)	3.1 (2.9)	6.1 (4.5)	4.3 (2.9)
Kaplan, 1990^107^	MPH vs. PBO	0.47 mg/kg	ADHD	6	6	13–16	49	Aggression in conners teacher rating scale	1.2 (0.7)		0.5 (0.7)	1.1 (0.6)
Klein, 1997^108^	MPH vs. PBO	Up to 60 mg/day	ADHD	37	37	6–15	35	Aggression in inattention/overactivity with aggression	10.5 (3.4)		6 (3.04)	8.3 (3.04)
Kolko, 1999^109^	MPH vs. PBO	0.3 or 0.6 mg/kg	ADHD	32	16	7–13	–	Overt aggression scale	–	–	0.35 (0.83)	1.1 (1.2)
Pelham, 1999^110^	MPH vs. PBO	0.3 mg/kg	ADHD	63	21	6–12	56	Oppositional/Defiant subscale in IOWA Conners rating scales	–	–	2.13 (1.67)	2.6 (2)
Pelham, 2005^111^	MPH vs. PBO	12.5, 25.0 or 37.5 cm^2^	ADHD	81	27	6–12	42	Oppositional/Defiant subscale in IOWA Conners rating scales	9.9 (2.53)		6.1 (4.0)	8.1 (4.2)
Pliszka, 2000^112^	MPH vs. PBO	25.2 × 13.1 mg/day	ADHD	20	18	8.1 ± 1.4	21	Aggression/Defiance subscale in inattention/overactivity with aggression	1.5 (1.0)	1.2 (1.1)	0.49 (0.73)	0.72 (0.95)
Posey, 2007^113^	MPH vs. PBO	0.25, 0.5, and 1 mg/kg/day	PDD, ASD	64	61	5–15	28	Hyperactive/Impulsive subscales in Swanson, Nolan, and Pelham Rating Scale-Revised	19.61 (4.22)		10.8 (5.99)	15.33 (5.81)
Shih, 2019^28^	MPH vs. Ato	M: 18–54 mg/day A: 0.5–1.2 mg/kg/day	ADHD	Ato: 80 MPH: 76	-	7–16	108	Aggressive behavior in Child Behavior Checklist	A: 62.75 (12.50) M: 64.98 (13.50)	–	A: 60.09 (10.42) M: 56.67 (10.28)	–
Sinzig, 2007^114^	MPH vs. PBO	20–30 kg: 20 mg 31–50 kg: 40 mg 50 kg: 60 mg	ADHD	43	42	6–16	28	Aggression in Fremdbeurteilungsbogen fur Storungen des Sozialverhaltens	0.69 (0.55)	0.59 (0.49)	0.43 (0.45)	0.54 (0.51)
Wolraich, 2001^115^	MPH vs. PBO	OROS: 18, 38 or 54 mg IR: 5, 10 or 15 mg	ADHD	192	90	6–12	28	Oppositional Defiant subscale in Swanson, Nolan, and Pelham Rating Scale-Revised	7.74 (4.20)	8.19 (3.8)	4.95 (3.87)	8.6 (4.82)
sNRI												
Arnold, 2006^116^	Ato vs. PBO	up to 1.4 mg/kg/day	ASD	16	16	5–15	42	Oppositional defiant factor in DSM-IV symptoms means	8.81 (5.67)	6.07 (3.83)	8.83 (6.67)	7.25 (6.34)
Bangs, 2008^32^	Ato vs. PBO	1.2 ± 0.28 mg/kg	ADHD	153	68	6–12	56	Oppositional Defiant subscale in Swanson, Nolan, and Pelham Rating Scale-Revised		18.9 (2.4)	15.2 (5.3)	16 (4.3)
Dittmann, 2011^117^	Ato vs. PBO	0.5 or 1.2 mg/kg	ADHD	60	59	6–17	63	Disruptive Behavior in Attention-Deficit and Disruptive Behavior Disorders Instrument	–	–	1.58 (0.46)	2.89 (0.6)
Dell’Agnello, 2009^33^	Ato vs. PBO	Up to 1.2 mg/kg/day	ADHD	105	32	6–15	56	Oppositional subscale in Conners’ Parent Rating Scale	11.7 (3.8)	12.2 (3.0)	10.5 (4.4)	13 (4.2)
Gau, 2007^118^	Ato vs. PBO	1.8 mg/kg	ADHD	69	29	6–16	42	Oppositional subscale in Conners’ Parent Rating Scale	–	–	9.9 (3.4)	11.6 (3.8)
Kelsey, 2004^34^	Ato vs. PBO	0.8–1.2 mg/day	ADHD	126	60	6–12	56	Restless-impulsive subscale in Conners’ Global Index Parent Evening	19.5 (6.8)	19.2 (5.9)	11 (7.7)	16.3 (7.5)
Kaplan, 2004^119^	Ato vs. PBO	2 mg/kg	ADHD, ODD	47	42	7–13	63	Hyperactive/Impulsive subscale in ADHD Rating Scale	20.3 (5.0)	19.7 (5.1)	12 (7.9)	16.1 (7.9)
Michelson, 2001^120^	Ato vs. PBO	0.5, 1.2 or 1.8 mg/kg/day	ADHD	213	83	8–18	56	Oppositional subscale in Conners’ Parent Rating Scale	10.17 (4.64)	9.1 (5.0)	7.64 (4.23)	8.5 (3.6)
Michelson, 2002^121^	Ato vs. PBO	0.5–0.75 mg/kg/ day	ADHD	84	83	6–16	42	Hyperactive/Impulsive in ADHD Rating Scale	15.7 (8.0)	15.3 (7.1)	10 (6.8)	13.2 (5.7)
Michelson, 2004^122^	Ato vs. PBO	1.2 mg/kg	ADHD	290	123	6–15	84	Oppositional subscale in Conners’ Parent Rating Scale	6.5 (4.4)	5.4 (4.2)	1.6 (4/9)	2.7 (4.3)
Montoya, 2009^123^	Ato vs. PBO	0.5–1.2 mg/kg/day	ADHD	100	51	10.3 (2.5)	84	Hyperactive/Impulsive in ADHD Rating Scale	17.6 (6.9)	17.3 (6.8)	11.9 (7.3)	15.2 (7.7)
Shih, 2019^28^	Ato vs MPH	M: 18–54 mg/day A: 0.5–1.2 mg/kg/day	ADHD	Ato: 80 MPH: 76	–	7–16	108	Aggressive behavior in Child Behavior Checklist	A: 62.75 (12.50) M: 64.98 (13.50)	–	A: 60.09 (10.42) M: 56.67 (10.28)	–
Weiss, 2005^124^	Ato vs. PBO	up to 1.8 mg/kg/day	ADHD	99	51	8–12	49	Oppositional subscale in Conners’ Parent Rating Scale	66.0 (14.9)	64.2 9 (15.0)	60.6 (14.9)	62.6 (11.1)
SDA												
Aman, 2004^125^	Ris vs. PBO	0.02–0.06 mg/kg	ODD, ADHD, CD	52	63	5–12	42	Conduct Problem in Nisonger Child Behavior Rating Form	32.9 (7.7)	34.5 (7.0)	17.7 (10.6)	28.3 (11.2)
Buitelaar, 2001^40^	Ris vs. PBO	1.5–4 mg/kg	CD, ODD, DBD	19	19	12–16	42	Modified Overt aggression Scale	11.5 (8.2)	9.0 (7.4)	6.7 (6.3)	8.1 (6.9)
Connor, 2008^38^	Que vs. PBO	50–800 mg	CD	9	10	12–17	49	Overt Aggression Scale	73.2 (34.3)	40.4 (23.8)	43.3 (55.6)	49.4 (27.8)
Findling, 2000^35^	Ris vs. PBO	0.25–3 mg/kg	CD	10	10	5–15	70	Rating of Aggression Against People and/or Property Scale	3.89 (1.27)	3.7 (1.71)	2.24 (1.33)	3.54 (1.77)
Findling, 2004^126^	Ris vs. PBO	1.51 mg/day	DBD	47	57	5–12	336	Conduct Problem in Nisonger Child Behavior Rating Form	32.3 (7.5)	34.5 (6.9)	17.3 (11.5)	27.7 (11.6)
LeBlanc, 2005^127^	Ris vs. PBO	0.01–0.06 mg/kg/day	CD, ODD	75	88	5–12	42	Aggression in Nisonger Child Behaviour Rating Form	10.1 (4.1)	10.6 (3.9)	4.5 (4.3)	8.3 (5)
Pandina, 2007^128^	Ris vs. PBO	0.01–0.06 mg/kg/day	ASD	26	28	5–12	56	Conduct Problem in Nisonger-Child Behavior Rating Form	17.2 (8.0)	6.5 (5.7)	21.5 (10.7)	15.5 (11.9)
Pavuluri, 2010^43^	Ris vs. Div	R: 0.5–2 mg/day D: 60–120 μg/mL	BD	Ris: 32 Div: 33	–	8–18	42	Overt aggression Scale	R: 13.36 (25.09) D: 16.16 (9.81)	–	R: 3.00 (3.64) D: 7.82 (7.18)	–
Razjouyan, 2018^129^	Ris vs. Ari	R: 0.25–1 mg/day A: 1.25–5 mg/day	ADHD	Ris:17 Ari:17	–	3–6	84	Conduct Problem in Conners’ Parent Rating Scale	R: 68 (10) Ar: 78 (13)	–	R: 51 (12) Ar: 59 (14)	–
Reys, 2006^130^	Ris vs. PBO	0.25–0.75 mg/day	DBD	172	163	5–17	84	Conduct Problem in Nisonger Child Behavior Rating Form	–	–	5 (9.5)	8.8 (11.2)
Shea, 2004^131^	Ris vs. PBO	0.01–0.06 mg/kg/day	ASD, PDD	40	39	5–12	56	Conduct Problem in Nisonger Child Behavior Rating Form	16.8 (9.4)	23.3 (12.0)	6.4 (974)	16.7 (9.5)
Safavi, 2016^132^	Ris vs. Ari	R: 0.25–2 mg/day A: 2.5–10 mg/day	ADHD	Ris: 20 Ari:20	–	3–6	56	Oppositional subscale in Conners’ Parent Rating Scale	R: 13.25 (4.25) Ar: 13.15 (3.01)	–	R: 10.18 (4.13) Ar: 9 (3.74)	–
Snyder, 2002^37^	Ris vs. PBO	0.40–3.80 mg/day	DBD	53	57	5–12	42	Conduct Problem in Nisonger Child Behavior Rating Form	33.4 (6.26)	32.6 (6.32)	17.6 (11.94)	25.8 (13.48)
Tohen, 2007^133^	Ola vs. PBO	2.5–20 mg/day	BD	100	52	13–17	21	Overt Aggression Scale	6.34 (3.67)	5.73 (2.94)	2.4 (2.6)	3.83 (2.1)
Lithium												
Carlson, 1992^134^	Li vs. PBO	–	CD	7	7	9–14	56	Aggression factor in Inpatient Global Rating Scale	50.3 (9.7)		50.3 (14.1)	47.8 (7.2)
Malone, 2000^39^	Li vs. PBO	300–2100 mg/day	CD	20	20	10–17	28	Overt aggression Scale	4.69 (2.43)	5.84 (2.58)	2.29 (2.65)	4.31 (4.26)
DRA												
Razjouyan, 2018^129^	Ari vs.Ris	A: 1.25–5 mg/day R: 0.25–1 mg/day	ADHD	Ris:17 Ari:17		3–6	84	Conduct Problem in Conners’ Parent Rating Scale	R: 68 (10) Ar: 78 (13)	–	R: 51 (12) Ar: 59 (14)	–
Safavi, 2016^132^	Ari vs. Ris	A: 2.5–10 mg/day R: 0.25–2 mg/day	ADHD	Ris: 20 Ari:20		3–6	56	Oppositional subscale in Conners’ Parent Rating Scale	R: 13.25 (4.25) Ar: 13.15 (3.01)	–	R: 10.18 (4.13) Ar: 9 (3.74)	–
Sallee, 2017^135^	Ari vs. PBO	10 mg/day if < 50 kg 20 mg/day if > 50 kg	Tourette’s Disorder	45	44	7–17	56	Hyperactive/Impulsive subscale in Swanson, Nolan, and Pelham-IV rating scale	1.1 (0.8)	1.1 (0.8)	0.52 (0.53)	0.75 (0.58)
SSRI												
Reddihough, 2019^136^	Flu vs. PBO	4–30 mg/day	OCD	49	43	7–18	112	Disruptiveness assessment	3.95 (2.24)	4.40 (2.16)	2.98 (2.14)	3.69 (2.09)
Potter, 2019^137^	Ser vs. PBO	2.5 mg/day or 5 mg/day	ASD	26	21	2–6	90	Aggression/ hyperarousal/hyperactivity in Visual Analog Scale	4.52 (2.51)	4.30 (2.49)	5.64 (2.71)	6.61 (2.46)
GRA												
Cueva, 1996^138^	Car vs. PBO	200–800 mg	CD	11	13	5–12	42	Aggression in Children's Psychiatric Rating Scale	5.88 (1.85)	5.79 (1.33)	3.08 (1.6)	3.18 (1.34)
Hellings, 2005^139^	VaS vs. PBO	75.5—77.8 mcg/mL	PDD	18	18	6–20	56	Parent Overt Aggression Scale	10.05 (8.25)	10.50 (11.91)	5.86 (3.84)	5.72 (4.62)
Hollander, 2010^42^	Div vs. PBO	minimum level 50 mg/ml	ASD	16	11	5–17	84	Overt aggression Scale	6.43 (1.41)	5.36 (2.2)	5.42 (2.17)	6.25 (1.28)
Pavuluri, 2010^43^	Div vs. Ris	R: 0.5–2 mg/day D: 60–120 μg/ml	BD	Ris: 32 Div: 33	–	8–18	42	Overt aggression Scale	R: 13.36 (25.09) D: 16.16 (9.81)	–	R: 2.81 (3.24) D: 5.82 (5.48)	–
SNRI												
Klein, 1992^140^	Imi vs. PBO	up to 200 mg/day	Anx	11	10	6–15	42	Conduct Problem in Conners’ Parent Questionnaire Scale	0.5 (0.52)		0.5 (0.24)	0.4 (0.15)
Klein, 1998^141^	Des vs. PBO	50–300 mg/day	MDD	16	15	13–18	42	Hostility in SCL-90	1.56 (1.0)	1.66 (1.1)	0.87 (0.7)	1.16 (0.7)
Spencer, 2002^142^	Des vs. PBO	3.4 mg/kg/day	ADHD, Tic	21	20	5–17	42	Hyperactive/Impulsive subscale in ADHD Rating Scale	–	–	12.78 (5.04)	21.56 (5.81)
SNDRI												
Findling, 2019^143^	Das vs. PBO	2 or 14 mg/day	ADHD	107	116	6–12	42	Hyperactive/Impulsive subscales in Conners’ Parent Rating Scale Conduct Problem in Nisonger Child Behavior Rating Form	82.7 (10.1)	83.5 (10.0)	71 (15.52)	74.3 (16.16)

*ADHD* Attention deficit hyperactivity disorder, *Anx* Anxiety disorder, *Ari* Aripiprazole, *ASD* Autism spectrum disorders, *Ato* Atomoxetine, *BD* Bipolar disorder, *Car* Carbamazepine, *CD* Conduct disorder, *DBD* Disruptive behavior disorders, *Das* Dasotraline, *Des* Desipramine, *Div* Divalproex, *Flu* Fluoxetine, *Imi* Imipramine, *IR* Immediate-release, *Li* Lithium, *MDD* Major depressive disorder, *MPH* Methylphenidate, *OCD* Obsessive–compulsive disorder, *ODD* Oppositional defiant disorder, *Ola* Olanzapine, *OROS* Oral dosage form, *PBO* Placebo, *PDD* Pervasive developmental disorder, *Que* Quetiapine, *Ris* Risperidone, *Ser* Sertraline, *VaS* Valproate sodium.

### Study characteristics

The characteristics of included studies are summarized in Table 1. Studies were performed and published between 1990 and 2021. Across studies, a total of 15 distinct psychotropic medications were represented. (1) one stimulant (Norepinephrine–dopamine reuptake inhibitor, NDRI): methylphenidate; (2) one non-stimulant ADHD medication (Selective norepinephrine reuptake inhibitors, SNRI): atomoxetine; (3) three second-generation antipsychotics (Serotonin dopamine antagonist, SDA): risperidone olanzapine and quetiapine (4) one dopamine receptor modulator (Dopamine receptor antagonist, DRA): aripiprazole (5) two SSRIs selective serotonin reuptake inhibitor): sertraline and fluoxetine; (6) three anticonvulsants (GABA receptor agonists, GRA): valproate sodium, divalproex, and carbamazepine (7) two TCAs (Serotonin-Norepinephrine Reuptake inhibitors, SNRI): desipramine and imipramine (8) one Serotonin-norepinephrine-dopamine reuptake inhibitor (Serotonin-norepinephrine-dopamine reuptake inhibitor, SNDRI): dasotraline (9) lithium.

A total of 9 diagnoses were included in the RCTs on disruptive behavior ADHD (n = 3988, 70.16%), disruptive behavior disorders (DBD) (n = 714, 12.56%), pervasive developmental disorder (n = 274, 4.82%), bipolar disorder (BD) (n = 217, 3.82%), ASD (n = 215, 3.78%), CD (n = 147, 2.59%), obsessive–compulsive disorder (n = 92, 1.62%), MDD (n = 31, 0.54%), and ODD (n = 6, 0.11%).

In total, 3490 and 2194 children and adolescents with psychiatric disorders were randomly assigned to active psychotropic medications and placebo, respectively. The age range of subjects in the RCT’s was 2–20 years (RCT’s mean = 7.03). Sixteen studies did not provide specific information on age. Subjects in both sets of RCT’s were predominantly male (83.75%) with 23 studies not reporting sex.

The mean duration of trials was 57.44 days (SD = 46.29) for medication treatment. There was one study that did not provide the duration.

### Network meta-analysis results for disruptive behavior

The relative efficacy of each agent group compared with placebo was conducted for disruptive behavior symptoms (Fig. 2b). The network consisted of 55 studies comparing nine agent groups versus placebo, which were stimulant (NDRI), non-stimulant ADHD medication (sNRI), second-generation antipsychotic (SDA), lithium, dopamine receptor modulator (DRA), SSRI, anticonvulsant (GRA), TCA (SNRI) and SNDRI.

Four agent groups were significantly more efficacious than placebo when data was combined in the NMA: second-generation antipsychotic with a Standardized Mean Differences (SMD) of 0.668 (95% CI 0.537–0.800), stimulant with 0.633 (95% CI 0.513–0.752), dopamine receptor modulator with 0.402 (95% CI 0.046–0.757) and non-stimulant ADHD medication with 0.386 (95% CI 0.280–0.492) (Fig. 2b). The effect sizes from traditional meta-analysis are presented in the supplementary data (Fig. S6).

Second-generation antipsychotics (SDA) had the highest probability for being the most efficacious agents for disruptive behavior symptoms (P score = 0.9356) followed by stimulants (P score = 0.8906), then dopamine receptor modulators (P score = 0.5932), while placebo ranked as the least efficacious in reducing the symptoms of disruptive behavior (P score = 0.0811) (Fig. 2c).

The Fig. 2d shows detailed results of pairwise meta-analyses. Even though the second-generation antipsychotics and stimulants showed the highest efficacies for disruptive behavior symptoms *when compared to placebo*, the direct and indirect pairwise comparisons *among medications* revealed they weren’t significantly more efficacious than various agent groups including TCA, lithium, SSRI and dopamine receptor modulator. There were significant differences between second-generation antipsychotic and anticonvulsant with an SMD of 0.574 (95% CI 0.216–0.933), between the second-generation antipsychotic and SNDRI with 0.460 (0.114–0.806), and between the second-generation antipsychotic and non-stimulant ADHD medication with 0.282 (0.114–0.451). Stimulant also showed significant differences in its efficacy when compared with anticonvulsant (SMD 0.539 95% CI 0.164–0.914), SNDRI (SMD 0.425, 95% CI 0.083–0.766) and non-stimulant ADHD medication (SMD 0.247, 95% CI 0.095–0.399).

### Quality assessment

We applied rigorous methods of quality assessment to provide evidence of the integrity of the NMA results. Supplementary Figs. S1 and S2 presented the methodological features examined for each trial and a summary result of the judged risk of bias across studies based on the Cochrane risk of bias tool. 22(40%) trials were assessed as a high risk of bias, 34 (61.81%) trials as unclear risk, and 12 (21.81%) as low risk of bias in disruptive symptoms.

Figure 2a show the network of eligible comparisons for efficacy in disruptive symptoms. All agent groups had at least one placebo-controlled trial. In network quality analysis for disruptive symptoms, we did not find evidence for heterogeneity (Q = 54.74, p = 0.1768, tau^2^ = 0.0086, I^2^ = 16). Also, there was no evidence of significant inconsistency between the direct and indirect estimates (i.e., all p-values were above 0.05) of disruptive symptoms (Tables S1, S2). The node-splitting method was also used to evaluate consistency between direct and indirect estimates (Fig. S3). For illustrating the consistency pattern that existed in each comparison, net heat plots were also formed to detect hot spots (red squares) indicating greater inconsistency among comparisons (Fig. S4). There was no significant inconsistency between direct and indirect evidence for most comparisons, as demonstrated by both the node splitting approach (P-value > 0.05) and net heat plots. Therefore, we concluded that the consistency model is valid in our NMA.

Potential publication bias in NMA was assessed through the funnel plots produced (Fig. S5). The result of the funnel plots showed no significant asymmetry pattern, and most studies were normally distributed in the funnel plot. Therefore, we determined that there was no significant publication bias in our research.

## Discussion

To our knowledge, this is the first NMA of Randomized Clinical Trials (RCTs) to date exploring the individual efficacy of pharmacological treatments on disruptive behavior problems (DBs) in youths with various psychiatric disorders. The efficacy of 9 psychotropic medications on treatment of symptoms representing underlying DBs varied widely. NMA revealed that, for reducing disruptive symptoms, second-generation antipsychotics, stimulant, and non-stimulant ADHD medications were significantly more efficacious than placebo, and second-generation antipsychotics were the most efficacious. For disruptive symptoms, there is a consistent finding that psychotropic medications with higher dopaminergic receptor affinity, including methylphenidate and risperidone, showed significant efficacy in reducing these symptoms compared to the other psychotropic agents^30,46,66^.

For reducing disruptive symptoms, the effect size of only second-generation antipsychotics (SMD = 0.668) was large enough to be compatible with treatment of other primary/secondary symptoms of, for example, schizophrenia^67^ or autistic spectrum disorder^68,69^ in youths. Stimulants (SMD = 0.633) also showed the efficacy for reducing disruptive symptoms, but the effect size was relatively small or similar compared to the impact on main symptoms of ADHD (SMD over 0.8 and 0.6 for hyperactivity and inattention)^70–72^. The effect sizes of the other agent groups were small to medium (SMD values of 0.3–0.5) for disruptive symptoms, and generally lower than the effect size found in previous meta-analyses for these agents in reducing primary symptoms of ADHD (non-stimulant ADHD medication: SMD over 0.7 and 0.5 for hyperactivity and inattention)^70–72^, Tourette’s disorder (dopamine receptor modulator: SMD over 0.5 for tic)^25,73^, and depression (SSRI: SMD over 0.5 for depression)^48,74^. Therefore, while our results show that stimulants, dopamine receptor modulators, SSRIs, and non-stimulant ADHD medications may all decrease disruptive symptoms and may have neural level impacts on the pathophysiology of disruptive behavior in youths with various psychiatric disorders, the magnitude of their effect might be lower than that found in treatment of primary symptoms of specific disorders.

The fact that psychotropic medications with dopaminergic affinity (i.e., second-generation antipsychotics, stimulants, and dopamine receptor modulators) were significantly efficacious for reducing disruptive behavior problems indicates that while the exact nature of dopamine’s relationship to disruptive behavior is not completely understood, dysfunction in the dopaminergic system itself may play a significant role in youths’ response to these medications (e.g. reducing affective aggression)^30,75,76^. Consideration should be given to the fact that although stimulants and second-generation antipsychotics both impact the dopaminergic system, their specific mechanisms of action are quite different (i.e., methylphenidate is a dopamine agonist, but risperidone and olanzapine are D_2_ receptor and 5-HT_2a_ antagonists).

A simplistic view of dopamine in mental disorders and their treatment has been that Dopamine levels are either too high or too low in specific brain regions. This may not, however, be sufficient to provide an explanation of the nature of dopaminergic dysfunction in various clinical presentations^77–81^. One potential explanation is that while stimulants and second-generation antipsychotics seem to have different mechanisms of action, they both result in augmenting *tonic* dopamine levels and reducing exaggerated *phasic* responses^79,81^. Secondly, it is also possible that not only the stimulants, but also the second-generation antipsychotics may directly affect top-down modulation by facilitating dopaminergic neurotransmission in the prefrontal cortex, even if they primarily affect the limbic system. It has been shown that acute treatment with second-generation antipsychotics, especially risperidone and olanzapine, increases the extracellular levels of dopamine in the prefrontal cortex (one of the main areas implicated in top-down modulation) as well as the nucleus accumbens^82,83^. The increased dopamine level in the nucleus accumbens induced by second-generation antipsychotics may mainly be due to the blockade of presynaptic D2 receptors^84^, since D2/3 receptor affinities of antipsychotics is associated with their preferential effects on dopamine levels there^82^. Since most prefrontal dopaminergic neurons do not possess D2 auto receptors, the increase dopamine release in the prefrontal cortex induced by second-generation antipsychotics appears to involve additional receptors^85^. Serotonergic pathways might also influence the regulation of extracellular dopamine in the prefrontal cortex^86^. Local activation of 5-HT2A receptors in the prefrontal cortex influences dopamine neuron activity in the ventral tegmental area and dopamine release in the mesocortical pathway^87^. The combined effects on D2 and 5-HT2A antagonism induced by second-generation antipsychotics (not just by reducing dopaminergic action) may have a more complex mechanism of reducing disruptive behavior in youths.

Clinically there has been concern that the effect of second-generation antipsychotics on disruptive symptoms is merely a product of their sedating effect^88^, due to their anti-histaminergic and anti-cholinergic actions^89^, however, it is also possible that these systems may play a critical role in disruptive behavior symptoms^90,91^. The fact that both stimulants and second-generation antipsychotics can exercise their impact on the neural areas of bottom-up emotional processing in addition to top-down cognitive control^92^, indicates that the symptoms of disruptive behavior may be driven by more complex mechanisms, which requires further future study.

In sum, the present NMA demonstrated that stimulants (i.e., methylphenidate) and second-generation antipsychotics, specifically risperidone, have large effect sizes in the treatment of DBs in children and youths, while mood stabilizers, SNRIs, α-2 agonists and antidepressants showed low to medium effect sizes. These results suggest that those agents with a higher affinity for dopamine receptors may be useful in the management of DBs, a result consistent with the hypothesis that DBs are due to deficits in the top-down inhibitory processes in the mesolimbic dopamine system.

There are a few caveats to offer. While this review examined various psychotropic medications for the treatment of symptoms of disruptive behavior, it did not take into account administration method, dosage or duration of treatment, safety or tolerability issues, or the age, gender and race/ethnicity of participants. These were not explored due to lack of reported information.

Secondly, we excluded studies that combined two or more psychotropic medications or psychotropic medication with other modalities of treatment (behavioral intervention/therapy) due to limitations of statistical analysis. We also did not examine studies that implemented neurobiological assessment methods to evaluate the neural level changes in the underlying pathophysiology of disruptive behavior (such as neuroimaging or genetics) to reduce measurement heterogeneity. Future study is warranted on these as well.

Third, some agent groups had small sample sizes, such as lithium (n = 27) and TCAs (SNRI) (n = 48). Previous work suggests that treatment efficacy would be overestimated in studies with small sample sizes^93^, therefore, it might be difficult to accurately assess the treatment efficacy of lithium and TCAs for DBs.

Fourth, because the symptom indicators for DBs applied in this study included symptoms in the context of externalizing disorders (i.e., ADHD, CD, ODD, DBD), there is a disparity in sample size between externalizing and internalizing disorders. The generalization of our findings to the full spectrum of pediatric psychiatric symptoms is therefore limited by the inability to interpret findings in symptoms manifested in internalizing disorders (i.e., depressive disorders, anxiety disorders, obsessive–compulsive and related disorders, trauma and stress-related disorders, and dissociative disorders). DBs in Autism Spectrum Disorder are a significant clinical issue, but these may or may not share the mechanisms of DBs in the externalizing disorders^94,95^. Thus, although in many cases stimulants and antipsychotic medications are being used for DBs in Autism Spectrum Disorders^94^, our current finding may not apply to those cases, and future study is necessary. In addition, disruptive behavior problems in other psychiatric diagnoses (such as BD and psychotic disorder) in children and adolescents may benefit from a separate and comprehensive study with similar design as this, since the underlying neurobiological mechanism of disruptive behavior problem in those diagnoses may significantly differ from the ones included in the current study^96,97^.

Lastly, including studies with various scales to measure DB symptoms may potentially limit interpretability. The analysis methodology we used (NMA), however, allows us to pool data from different trials with similar treatment arms, to measure relative effects of interventions^98^. In addition, all of the studies that were included in the NMA implemented the scales to measure similar if not identical disruptive behavior elements, such as oppositional/defiance, overt aggression, and hyperactivity/impulsivity (see Table 2)^99^. Previous studies demonstrated high levels of overlap and strong correlations of these symptoms with each other^100^.

Despite these limitations, our study was able to identify the efficacy of various psychotropic medications for treating the various symptoms of disruptive behavior, and speculates that this efficacy may be related to the mechanism of action of medications at the neural level. In particular, psychotropic medications with dopaminergic affinity, especially the second-generation antipsychotics, were particularly efficacious in treating the symptoms of disruptive behavior.

## Clinical implication and conclusion

Based on this review, several pharmacotherapies such as second-generation antipsychotics and stimulants may have potential efficacy for the treatment of disruptive behavior symptoms in the psychiatric diagnoses included in our study. This may be a first step toward refining and individualizing treatment options for youth with externalizing disorders, as well as eventually improving our understanding of the underlying neurobiological mechanisms of disruptive behavior in these diagnoses. Future study may consider incorporating a trans-diagnostic concept into designing RCTs targeting DBs.

### Supplementary Information

Supplementary Information.

## Data Availability

The data that support the findings of this study are available from the corresponding author, upon reasonable request.

## Abbreviations

ADHD: Attention deficit hyperactivity disorder
ANX: Anxiety disorder
ASD: Autism spectrum disorder
BD: Bipolar disorder
CD: Conduct disorder
DBD: Disruptive behavior disorder
DB: Disruptive behavior
DRA: Dopamine receptor antagonist/dopamine receptor modulator
GRA: GABA receptor agonists
MDD: Major depressive disorder
NDRI: Norepinephrine–dopamine reuptake inhibitor/stimulant
NMA: Network meta-analysis
OCD: Obsessive–compulsive disorder
ODD: Oppositional defiant disorder
PDD: Pervasive developmental disorder
RCT: Random control trial
SDA: Serotonin dopamine antagonist/second-generation antipsychotics
SMD: Standardized mean differences
SNDRI: Serotonin-norepinephrine-dopamine reuptake inhibitor
sNRI: Selective norepinephrine reuptake inhibitors/non-stimulant ADHD medication
SNRI: Serotonin-norepinephrine reuptake inhibitors/tricyclic antidepressants
SSRI: Selective serotonin reuptake inhibitor
