# Author Correction: Psychopharmacological treatment of disruptive behavior in youths: systematic review and network meta-analysis

**DOI:** 10.1038/s41598-023-51047-7

**Published:** 2024-01-09

**Authors:** Ji-Woo Seok, Brigette Soltis-Vaughan, Brandon J. Lew, Aatiya Ahmad, R. J. R. Blair, Soonjo Hwang

**Affiliations:** 1 Department of Psychiatry, University of Nebraska Medical Center, 985578 Nebraska Medical Center, Omaha, NE 68198-5578 USA; 2 Digital Health Research Division, Korea Institute of Oriental Medicine, Daejeon, South Korea; 3 Child and Adolescent Mental Health Centre, Mental Health Services, Capital Region of Denmark, Copenhagen, Denmark

Correction to: *Scientific Reports* 10.1038/s41598-023-33979-2, published online 28 April 2023

The original version of this Article omitted an affiliation for Ji-Woo Seok. The correct affiliations are listed below.

1. “Department of Psychiatry, University of Nebraska Medical Center, 985578 Nebraska Medical Center, Omaha, NE, 68198–5578, USA”
2. “Digital Health Research Division, Korea Institute of Oriental Medicine, Daejeon, South Korea”

The original Article has been corrected.

